# Effect of eplerenone on parathyroid hormone levels in patients with primary hyperparathyroidism: a randomized, double-blind, placebo-controlled trial

**DOI:** 10.1186/1472-6823-12-19

**Published:** 2012-09-13

**Authors:** Andreas Tomaschitz, Astrid Fahrleitner-Pammer, Burkert Pieske, Nicolas Verheyen, Karin Amrein, Eberhard Ritz, Katharina Kienreich, Jörg H Horina, Albrecht Schmidt, Elisabeth Kraigher-Krainer, Caterina Colantonio, Andreas Meinitzer, Stefan Pilz

**Affiliations:** 1 Department of Cardiology, Medical University of Graz, Auenbruggerplatz 15, Graz, 8036, Austria; 2 Department of Internal Medicine, Division of Endocrinology and Metabolism, Medical University of Graz, Auenbruggerplatz 15, Graz, 8036, Austria; 3 Department of Medicine, Division of Nephrology, University Hospital Heidelberg, Heidelberg, Germany; 4 Department of Internal Medicine, Division of Nephrology, Medical University of Graz, Graz, Austria; 5 Clinical Institute of Medical and Chemical Laboratory Diagnostics, Medical University of Graz, Graz, Austria; 6 Department of Epidemiology and Biostatistics and EMGO Institute for Health and Care Research, VU University Medical Center, Amsterdam, The Netherlands

**Keywords:** Aldosterone, Mineralocorticoid receptor blocker, Hyperparathyroidism

## Abstract

**Background:**

Increasing evidence suggests the bidirectional interplay between parathyroid hormone and aldosterone as an important mechanism behind the increased risk of cardiovascular damage and bone disease observed in primary hyperparathyroidism. Our primary object is to assess the efficacy of the mineralocorticoid receptor-blocker eplerenone to reduce parathyroid hormone secretion in patients with parathyroid hormone excess.

**Methods/design:**

Overall, 110 adult male and female patients with primary hyperparathyroidism will be randomly assigned to eplerenone (25 mg once daily for 4 weeks and 4 weeks with 50 mg once daily after dose titration] or placebo, over eight weeks. Each participant will undergo detailed clinical assessment, including anthropometric evaluation, 24-h ambulatory arterial blood pressure monitoring, echocardiography, kidney function and detailed laboratory determination of biomarkers of bone metabolism and cardiovascular disease.

The study comprises the following exploratory endpoints: mean change from baseline to week eight in (1) parathyroid hormone(1–84) as the primary endpoint and (2) 24-h systolic and diastolic ambulatory blood pressure levels, NT-pro-BNP, biomarkers of bone metabolism, 24-h urinary protein/albumin excretion and echocardiographic parameters reflecting systolic and diastolic function as well as cardiac dimensions, as secondary endpoints.

**Discussion:**

In view of the reciprocal interaction between aldosterone and parathyroid hormone and the potentially ensuing target organ damage, the EPATH trial is designed to determine whether eplerenone, compared to placebo, will effectively impact on parathyroid hormone secretion and improve cardiovascular, renal and bone health in patients with primary hyperparathyroidism.

**Trial registration:**

ISRCTN33941607

## Background

Parathyroid hormone (PTH) is synthesized and secreted in the chief cells in the parathyroid gland mainly in response to a decreased circulating ionized calcium concentration. PTH regulates the calcium und phosphate homeostasis by activating osteoclasts and osteoblasts, enhancing intestinal Ca^2+^ absorption, promoting the synthesis of active vitamin D in the kidney and increasing active renal Ca^2+^ reabsorption. Elevation of plasma Ca^2+^ concentration in turn decreases PTH secretion by activating calcium sensing receptors located on chief cells. A well-balanced calcium homeostasis is crucial for the regulation of cell signalling, neuromuscular function and bone metabolism.

Primary hyperparathyroidism (PHPT), the third most common endocrine disorder, is characterized by excess PTH secretion, inappropriate with respect to the prevailing circulating ionized calcium concentration [1]. The identification of PTH receptors within the cardiovascular (CV) system e.g. in cardiomyocytes, vascular smooth-muscle and endothelial cells, indicates that PTH excess may have a potential impact on CV health. In fact, various observational studies linked PTH excess to a higher risk of hypertension, left-ventricular hypertrophy, arrhythmia and metabolic disorders [2-4].

Several observational studies in humans point to an eminent role of the mineralocorticoid hormone aldosterone, produced within the zona glomerulosa (ZG) of the adrenal gland, in the pathogenesis of CV and renal disease [5-7]. Relative excess of aldosterone may play an important role in the genesis of CV damage even in the absence of primary aldosteronism [8].

Accumulating evidence points to the bidirectional interplay between PTH and aldosterone as an important mechanism behind the increased risk of CV damage observed in PHPT [9,10]. Experimental and clinical data support the notion that PTH directly stimulates adrenal steroid secretion by inducing calcium entry in adrenal ZG cells via binding to PTH/PTH-related protein receptor (PTH/PTHrP receptor = PTH1R), voltage-gated L-type calcium channels and by activating various signal transduction pathways [11,12]. The resulting relative aldosterone excess triggers increased PTH secretion by facilitating renal and fecal calcium loss, which in turn aggravates PTH secretion and CV damage [13].

Given the linkage between aldosterone and PTH, treatment of either disease (primary aldosteronism and PHPT) results in positive effects in the other hormone system. However, studies evaluating the effects of mineralocorticoid receptor (MR)-blockade on PTH secretion, CV health and bone metabolism in patients with PHPT are missing. We therefore suggested the working hypothesis that in patients with PHPT MR-blockade with eplerenone decreases iPTH(1–84) levels and exerts beneficial effects on CV and bone health. To this end we propose a randomized controlled trial to test this hypothesis in PHPT patients.

## Methods/design

### Study design, endpoints and safety

EPATH is a single-center, double-blind, placebo-controlled, randomized, parallel group trial. Patients with PHPT diagnosed according to the report of the 3rd International Workshop on Diagnosis of PHPT will be screened for inclusion into the study [14]. Patients diagnosed with symptomatic/asymptomatic PHPT will be consented and screened for eligibility to participate in the EPATH trial. Overall 110 patients will be randomized to receive eplerenone or placebo in a 1:1 ratio (55 patients will receive eplerenone and 55 patients will receive placebo).

The design, conduction and reporting of the EPATH study adheres to the recommendations of the CONSORT Statement ( http://www.consort-statement.org/). The study will be performed in accordance with the Good Clinical Practice (GCP) guidelines and the Declaration of Helsinki. The study has been approved (N° 24–032 ex 11/12; EudraCT number: 2011-005683-21) by the Ethics Committee of the Medical University of Graz, Austria.

The primary endpoint of the study is to evaluate the efficacy of eplerenone relative to placebo to reduce PTH(1–84) concentration, measured by two different laboratory methods, in patients with PHPT. Secondary endpoints are the mean changes from baseline after 8-weeks with eplerenone versus placebo, in the following measurements: (1) 24-hour systolic and diastolic ambulatory blood pressure (ABP) levels; (2) biomakers of CVD; (3) biomarkers of bone metabolism: osteocalcin, β-crosslaps, bone alkaline phosphatase and tartrate-resistant acid phosphatase; (4) 24-h urinary albumin/protien excretion; and (5) echocardiographic parameters related to systolic and diastolic function as well as cardiac dimensions (i.e. left ventricular ejection fraction, E/E´ ratio, left ventricular end-diastolic/systolic pressure/volume/index and LV posterior wall thickness).

We decided to evaluate the effect of MR-blocker eplerenone on iPTH(1–84) - the primary outcome – and on CV and bone health – the secondary outcomes - after 8 weeks because (1) preliminary data from the Graz Endocrine Causes of Hypertension (GECOH) study suggest that MR-blockade with eplerenone might exert “rapid” effects on iPTH(1–84) levels [15]; (2) eplerenone has been shown to significantly lower BP after a few days of treatment, indicating that this dose may be sufficient to impact on iPTH(1–84) levels [16]; and (3) in order to minimize the drop-out number due to eplerenone’s higher selectivity for the MR, which yields a superior tolerability profile in terms of sexual side effects, compared to spironolactone. In addition, findings from previous studies suggested that eplerenone appears to produce more consistent inhibition of some of the nongenomic effects of aldosterone than spironolactone [17]. Finally, it has been speculated that the extended half-life of the active metabolites of spironolactone increase the risk of hyperkalemia and its associated complications [18]. Conversely, the relatively short half-life of eplerenone and lack of inactive metabolites may decrease the risk of hyperkalemia.

Considering the higher prevalence of arterial hypertension and cardiovascular disease (CVD) in PHPT patients, which is suggested to result in part from a reciprocal interaction between aldosterone and PTH, MR-blockade might be a promising therapeutic strategy to decrease risk of the development and progression of CVD even in normotensive patients without materially increasing the risk of hypotensive side effects [19,20]. In case of severe side effects related to hypotension, the study drug however will be discontinued.

**Figure 1:**
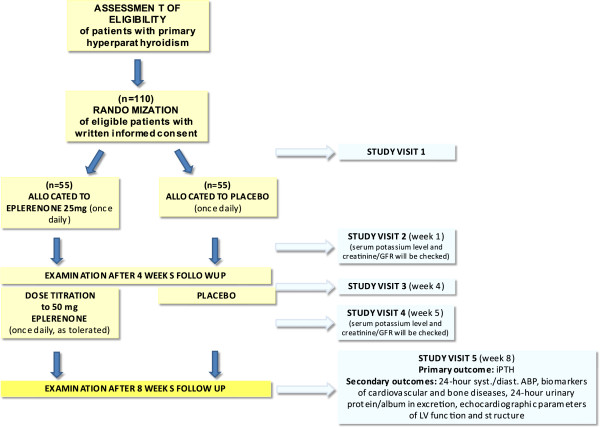
Flow chart for the EPATH trial.

The study schema and visit schedule are illustrated in flow chart in Figure 1. The preliminary screening for study eligibility is based on medical records performed by the study staff at or before the baseline visit. A written informed consent will be provided by each eligible participant, who will be randomly assigned in a double blind fashion to receive eplerenone 25 mg once daily (given in the morning) or a matching placebo. If no adverse effects of eplerenone treatment with 25 mg once daily will be observed after 4 weeks, the dose will be up-titrated by protocol to the target dose of 50 mg once daily. This dose titration strategy was chosen to minimise the risk of adverse effects, in particular hyperkalemia. At week 1 and each subsequent clinic visit, the serum potassium and creatinie level will be checked. Overall, the study consists of an 8-week, double-blind, randomized (active) treatment period.

Patients will be requested to be fasting and abstain from tea, coffee and smoking for overnight at least 12 hours before applying at the study outpatient. Intake of any drug should be avoided in the morning before blood collection. Investigations will be done in the morning (8:00 to 12:00). Extreme care will be taken to avoid haemolysis and prolonged stasis. Laboratory surveillance of serum electrolytes (potassium, sodium, magnesium and calcium) and renal function (serum creatinine and estimated glomerular filtration rate (GFR) according to the MDRD formula) is required at each study visit. Immediate suspension of the study drug eplerenone will be disposed for any measured serum potassium ≥ 5.5 mmol/L, and a down-titration of the study drug will be initiated for any measured serum potassium ≥ 5.0 mmol/L. Furthermore, the study drug will be discontinued in case of a rise of serum creatinine ≥ 3.0 mg/dL or GFR < 30 mL/min (or downtitration between GFR 30-49 mL/min), severe anaphylactic reactions following or intolerance due to eplerenone intake, requirement for an open-label use of eplerenone or potassium sparing diuretics, or formal withdrawal of the consent.

To minimise the risk of adverse events (e.g. hyperkalemia) patients with an eGFR ≤ 50 mL/min and/or a serum potassium > 5 mmol/L will be excluded from enrollment into the EPATH Study. Furthermore, the dose of 25 mg eplerenone once daily is comparably low and will be titrated to 50 mg once daily, if no adverse effects have been observed and if well tolerated.

If women of child-bearing age participate in the EPATH study, pregnancy will be ruled out at the beginning of the active participation before study enrolment. Further pregnancy tests will be performed monthly in our outpatients (spot urine test), and additionally as a self-test at home (after thorough education and instruction to perform test self-tests and after notification per telephone). Female participants of child-bearing age will be instructed to perform contraception throughout the active study period.

Because MR-blocker treatment is currently recommended in all patients with persisting symptoms (NYHA class II–IV) and an EF <35%, despite treatment with an ACE inhibitor (or an ARB) and a beta-blocker, special care was taken to avoid serious side effects such as hyperkalemia in PHPT patients by (1) excluding those participants from study participation, who are at higher risk of developing hyperkalemia; (2) a dose titration strategy; (3) monitoring of serum electrolytes and renal function at each study visit [21].

Randomization will be done with assistance of the Institute of Medical Informatics, Statistics and Documentation of the Medical University of Graz (http://www.randomizer.at). The study staff and participants will be blinded to treatment assignment for the duration of the study.

Study medication of either eplerenone (Pfizer Corporation Austria, Vienna, Austria) or placebo will be packed in numbered pill boxes (identical in appearance and packages) according to both a computer generated randomization list and to international standards, respectively. This will be done by qualified staff at the pharmacy of the Medical University of Graz, which has extensive experience with placebo controlled trials.

### Setting

Eligible study participants will be recruited from the outpatient clinic of the Department of Internal Medicine, Division of Endocrinology and Metabolism and Division of Cardiology, Medical University of Graz, Austria. All baseline and follow-up visits will be conducted at the Department of Internal Medicine, Division of Endocrinology and Metabolism and Division of Cardiology and at the Center of Medical Research of the Medical University of Graz, Austria.

### Inclusion and exclusion criteria

Eligible patients will be men and women of at least 18 years of age with PHPT, who (1) do not meet criteria for surgical treatment; or (2) in whom surgery is recommended, but not performed because of patient and/or physician preference; or (3) perceived medical contraindications [22].

Exclusion criteria include (1) 25(OH)D levels <20 ng/dl (50 nmol/liter); (2) estimated GFR (according to the MDRD formula) ≤ 50 ml/min; (3) serum potassium > 5.0 mEq/L (mmol/L) at baseline or > 5.5 mEq/L (mmol/L) during active study period; (4) side effects related to hypotension; (5) pregnancy or lactating women; (6) drug intake as part of another clinical study 4 weeks before enrolment into the EPATH study and/or during the active study period; (7) any disease with an estimated life expectancy below 1 year; (8) chemotherapy or radiation therapy during the study; (9) intolerance to eplerenone or any ingredient occurring in eplerenone; (10) severe acute or chronic liver diseases (Child-Pugh Class C); and (11) concurrent intake of potassium sparing drugs, e.g. diuretics (amiloride and triamterene) or CPY3A4-inhibitors and ongoing potassium supplementation.

### Measurements

#### Anthropometry

Weight and height will be measured wearing no shoes and light clothes and waist and hip ratio will be determined according to published recommendations.

#### Laboratory measurements

All blood samples will be centrifuged within 1 hour after sampling and will be measured at latest 4 hours after blood collection. Before analysis or freezing all samples will be kept at room temperature, except of the samples for determination of iPTH(1–84) (and plasma aldosterone), which will be kept at 4° Celsius. Remaining samples for long-term storage (whole blood, serum, plasma, urine and saliva samples) will be kept at −70° to 80° Celsius at the Biobank Institute of the Medical University of Graz.

Measurement of iPTH(1–84) (pg/mL) will be performed by electrochemiluminiscence immunoassay “ECLIA” (Elecsys immunoassay analyzer, Cobas®, Roche Diagnostics GmbH, Mannheim, Germany). The Elecsys assay for determining intact PTH employs a sandwich test principle in which a biotinylated monoclonal antibody reacts with the N-terminal fragment (1–37) and a monoclonal antibody labeled with a ruthenium complex reacts with the C-terminal fragment (38–84). The antibodies used in this assay are reactive with epitopes in the amino acid regions 26–32 and 37–42. Blood for iPTH(1–84) determination will be collected with standard EDTA plasma tubes and centrifuged within 5 to 10 minutes.

[Conversion factors of iPTH(1–84) are: pg/mL × 0.106 = pmol/L and pmol/L × 9.43 = pg/mL, respectively. Measuring range of the assay: 1.20 (=lower detection limit) to 5000 pg/mL (=maximum of the master curve); Reference range: 15–65 pg/mL, interassay coefficient of variation 3.0-6.5% for PTH 26.7-261 pg/mL).

PTH will be further determined using the electrochemiluminiscence LIAISON N-tact PTH immunoassay (DiaSorin Inc., Stillwater, MN, USA).

The measurements of further parameters (25(OH)D, osteocalcin, β-CrossLaps, bone alkaline phosphatase, tartrate-resistant acid phosphatase, osteoprotegerin, NT-pro-BNP) have been previously described in detail [15,23].

All other laboratory measurements will be performed according to current routine laboratory methods at the Medical University of Graz.

Measurement of omics-based biomarkers will be performed by Liquid chromatography–mass spectrometry at the Clinical Institute of Medical and Chemical Laboratory Diagnostics, Medical University of Graz [24].

#### Genetic investigations

Genetic analyses (e.g. PCR) will be performed in order to investigate the impact of e.g. polymorphisms of the calcium-sensing receptor or of the aldosterone synthase (CYP11B2) on PTH- and aldosterone secretion. Genome-wide association analyses will be performed - within the limits of a hypothesis testing strategy - in order to examine novel genes/loci implicated in the regulation of aldosterone and PTH synthesis in concert with larger studies. Genetic testing will be further initiated in suspicion of familial form of hyperparathyroidism. Emphasis will be placed upon the genes associated with familial hypocalciuric hypercalcemia (FHH), multiple endocrine neoplasia syndrome type I (MEN1), MEN2, and the hyperparathyroidism jaw-tumor syndrome.

#### Echocardiography

Comprehensive echocardiographic analysis of cardiac function and dimensions will be performed by experienced physicians on echocardiographic device (GE Vivid 3® Pro Echocardiography System, United Kingdom) according to current guidelines of the American Society of Echocardiography. Tissue Doppler indices will be recorded at the septal and lateral base of the mitral annulus.

#### 24-hour systolic/diastolic ambulatory blood pressure monitoring (ABPM)

Our stuff will be carefully trained to ensure the accuracy of the ABPM measurement. The ABPM measurements will be performed in accordance to the established protocols of the European Society of Hypertension and the American Association for Medical Instrumentation. Measurements of 24-hour ABP will be performed by a validated ABP monitor (Spacelabs 90217; Spacelabs Healthcare GmbH) and mean 24-hour systolic and diastolic ABP will be recorded. A minimum of 14 readings during the day and 7 readings overnight will be required for valid 24-hour ABPM.

#### Questionnaires

The validated Short Form 36 questionnaire (SF-36)12 will be used as a general estimate for qualtity of life. The Kansas City Cardiomyopathy Questionnaire is a 23-item, self-administered instrument, which will be used to quantify physical function, symptoms (frequency, severity and recent change), social function, self-efficacy and knowledge, and quality of life.

### Statistical methods

#### Sample size calculation

Sample size calculation for the primary endpoint iPTH(1–84) is based on (1) an observed effects of eplerenone treatment (and adrenalectomy) on iPTH(1–84) levels in a previously published study [25]; (2) the proven significant association between (elevated) PTH levels on risk of fatal cardiovascular events in humans [3]; and (3) data derived from patients, which have been managed at the outpatient clinic of the Division of Endocrinology and Metabolism at the Medical University of Graz, with primary hyperparathyroidism.

Based on the observational data from the GECOH study, MR-blockade (by eplerenone and spironolactone) in patients with primary aldosteronism was associated with an approximate 10.0 pg/mL decrease in PTH levels after a mean follow-up of 12 months [25]. However, two patients at short-term follow-up of 4 weeks revealed a similar decrease of PTH levels with MR-blockade compared to long-term follow-up, indicating a rapid effect of MR-blockade on PTH levels. We are, however, aware that due to limited evidence regarding the effects of eplerenone in patients with PHPT, final conclusions cannot be drawn.

We further evaluated whether an increase of 10.0 pg/mL PTH is translated into a significant higher risk of CV mortality. In the LURIC study comprising 3.300 patients referred to coronary angiography the increment of 10 pg/mL PTH was significantly related to a 4% [Hazard Ratio (95% CI): 1.04 (1.03-1.06) p < 0.001] higher risk of cardiovascular death [3]. We assumed an effect size of 10.0 pg/mL, assuring sufficient power to decrease PTH by eplerenone 25/50 mg once daily. Thus, for a two-sided alternative-hypothesis with an α of 0.05 and a power (1-β) of 80% we calculated a sample size of 51 study participants per group. To compensate for drop-outs during the study (estimated to around 10%) we plan to enrol 55 patients per group.

#### Outcomes

Outcome variables will be tested for normal distribution by use of the Kolmogorov-Smirnov test and by further descriptive statistics. Normally distributed continuous variables will be given as mean [with standard deviation (SD)], variables with skewed distribution as median with interquartile range, and categorical variables as percentages. For parametric procedures all skewed distributed continuous parameters will be logarithmically transformed (log10). Analysis of covariance, adjusted for the respective baseline values, will be used to analyze the continuous primary and secondary outcome variables.

All statistical tests to assess the treatment difference between eplerenone and placebo will be performed using a 2-sided hypothesis test at 5% significance level. Data will be analysed using SPSS 17.0 statistical package (SPSS, Inc., Chicago, IL, USA).

## Discussion

In view of the potential interrelationship between aldosterone and PTH in patients with PHPT we hypothesize that the inhibition of aldosterone mediated effects by MR-blockade with eplerenone in PHPT patients not undergoing surgery will (1) result in a decrease of PTH(1–84) levels, (2) lead to beneficial effects on bone metabolism and (3) exerts beneficial effect on CV health and kidney function. Thus, the EPATH trial is the first clinical trial to specifically examine the effects of MR-blockade on PTH levels, bone, renal and CV health in patients with PTPH not undergoing surgery.

The “Third International Workshop on Asymptomatic Primary Hyperparathyroidism” concluded that further studies are definitely warranted in those patients with PHPT in whom surgery is not recommended (or indicated) [26]. This is of particular importance considering that even asymptomatic PHPT is associated with higher CV morbidity and mortality. To date, little is known about the mutual interaction between these two hormones and its potential role for target organ damage. In experimental rat studies combined 5/6 nephrectomy and parathyroidectomy resulted in decreased aldosterone levels compared to control rats [27]. Studies in PHPT patients noted markedly decreased plasma aldosterone levels and plasma renin activity after parathyroidectomy [28,29]. In a larger study, Brunaud et al. reported in patients with PTH excess after parathyroidectomy significantly decreased aldosterone and BP levels [30].

Recently, Maniero et al. impressively demonstrated the expression of the MR in both PTH-secreting adenoma and in normal parathyroid gland tissue [31]. Interestingly, the MR was predominantly located in the nucleus of the parathyroid cells, indicating that aldosterone (and cortisol), participate in a “tonic” regulation of PTH synthesis and secretion. It is one crucial aim of the EPATH trial to evaluate whether eplerenone inhibits the modulation of PTH secretion and synthesis of aldosterone and cortisol. In fact, cortisol is also suggested to activate the MR, particularly in conditions of an increased reactive oxygen species generation [32]. Moreover, plasma cortisol levels increase after PTH infusion and cortisol is suggested to participate in the regulation of PTHrP expression [33,34]. In addition, we cannot exclude the possibility that MR blockade can directly inhibit PTH secretion independent of aldosterone and cortisol. Finally, it is unclear whether eplerenone inhibits some of the non genomic effects of aldosterone (and cortisol) [17].

**Figure 2:**
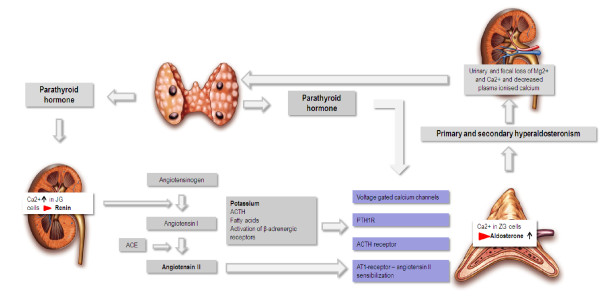
**Overview of the bidirectional interplay between aldosterone and PTH.** Calcium influx is crucial for activating the synthesis of renin in juxta glomerular cells and aldosterone in zona glomerulosa cells. Renal renin synthesis is mainly controlled by tubular sodium concentration, arterial blood pressure and the sympathetic nervous system. PTH is suggested to stimulate renin synthesis by increasing calcium levels in JG cells. Extracellular potassium, adrenocorticotropic hormone and angiotensin II are major stimulators of aldosterone synthesis in the adrenal glands. Both factors interact with voltage-gated calcium channels and depolarize the zona glomerulosa cells, which results in elevated intracellular calcium levels. PTH might directly stimulate aldosterone synthesis by binding to the PTH1R and adrenocorticotropic hormone receptor. In addition, PTH is suggested to increase sensibilization of angiotensin II, which by itself reduces cellular calcium extrusion through activating Na+/Ca–exchangers in zona glomerulosa cells. Aldosterone in turn facilitates renal and fecal calcium loss resulting in further PTH secretion.

Findings from previous studies support the notion that PTH may stimulate adrenal aldosterone synthesis directly. The identification of the PTH/PTH-RP receptor (PTH1R) in human and rat adrenal cortex binds intact PTH and the biologically active N-terminal fragment PTH 1–34 and provides an indication for direct effects of PTH on ZG cells [35]. Mazzocchi et al. and others found that PTH and PTH-related protein increase in human adrenals aldosterone production by binding to the PTH/PTH-RP receptor [11,12]. In isolated rat ZG cells exposed to PTH(1–84) and PTH(1–34) Olgaard et al. observed a 2-fold increased of aldosterone release resulting from PTH induced Ca^2+^ionophore-like effects [36]. Thus, PTH might directly mediate aldosterone production by inducing calcium entry in adrenal ZG cells via binding to PTH/PTH-RP receptors, voltage-gated L-type calcium channels and by activating various signal transduction pathways and indirectly by activating the renin–angiotensin system. Figure 2 summarizes the suggested pathways of an interplay between PTH and the renin-angiotensin-aldosterone system. With the EPATH trial we aim to evaluate whether the blockade of aldosterone by eplerenone breaks through the stimulating effects of PTH on aldosterone secretion by decreasing aldosterone mediated renal calcium loss and subsequently enhanced PTH secretion. 

Several cross-sectional and prospective studies documented a strong relationship between both elevated PTH and aldosterone levels, respectively and arterial hypertension as well as increased arterial stiffness [37-40]. Accordingly, one might speculate that the interplay between both hormones aggravates BP elevation, and remodeling of blood vessels in patients with elevated PTH [41]. This hypothesis would be in line with the observation of Morfis et al. that aldosterone levels are strongly related to systolic and diastolic ABPM values. However, the correlation is less significant if PTH is considered as confounder [42]. In addition, PTH mediated intracellular and mitochondrial calcium overload results in a disturbed redox status in various tissues i.e. in cardiac myocytes, resulting in increased oxidative stress [43]. These mechanisms might in part explain the significant relationship between circulating aldosterone and PTH levels and higher risk of left ventricular hypertrophy and sudden cardiac death in patients at high CV risk [3,8,44]. Importantly, aldosterone induced urinary and fecal Ca^2+^ and Mg^2+^ excretion was attenuated by spironolactone. Furthermore, intake of MR-blockade resulted in reduced intracellular calcium overload and improved redox status in peripheral blood mononuclear cells [13]. The above data suggest that through its calcium wasting properties aldosterone might activate the PTH-cascade resulting in further target organ damage, which might be reversed by MR-blockade. In the EPATH study we aim to evaluate whether MR-blockers with eplerenone has effects on 24-h ambulatory arterial blood pressure, NT-pBNP, kidney function, LV function and structure in patients with PTH excess.

Increased aldosterone mediated renal calcium loss might be the key mechanisms for the subsequent development of hyperparathyroidism in chronic heart failure (HF). The majority of studies demonstrated calcium wasting properties of aldosterone, independent of PTH, particularly in the setting of dietary salt excess [45,46]. In rats treatment with aldosterone/1% NaCl causes increased urinary and fecal Ca^2+^ and Mg^2+^ excretion, hypocalcemia, hypomagnesemia and consequently secondary hyperparathyroidism, as well as increased tissue calcium concentration. Due to increased PTH activity bone mineral density and strength were significantly reduced. Accordingly, HF patients suffering from secondary aldosteronism and PTH excess are at significantly higher risk of orthopedic fractures, in particular hip fracture, compared to controls [47]. In contrast, treatment with the MR-blocker spironolactone prevented reduction of bone mineral density in aldosterone/salt/loop diuretics treated rats [48]. However, studies in humans that evaluated the association between MR-blocker use with fracture risk are rare. One study documented that spironolactone use was inversely associated with total fracture risk (odds ratio: 0.575; 95% CI: 0.346 to 0.955, p = 0.0324) in men with congestive HF [49]. Treatment with the MR-blocker spironolactone was inversely related to fractures in chronic HF males. It remains (however) to be determined by adequately designed placebo controlled randomized trials whether MR-blockade affects bone metabolism and fracture risk in various risk groups. In the EPATH trial we attempt to evaluate the effects of aldosterone on bone metabolism reflected by laboratory markers of bone metabolism in patients at PTH excess. Considering the above mentioned evidence, interventional studies are urgently needed to evaluate the effects of MR-blockade on bone mineral density and bone metabolism in chronic HF patients.

The majority of experimental animal and human studies identified a clinically relevant interplay between aldosterone and PTH levels. The bidirectional stimulatory effects of PTH and aldosterone may potentiate the risks of development and progression CV and bone, kidney disease in patients with PHPT. It has been suggested that treatment of either disease, aldosterone excess and hyperparathyroidism, might positively affect the CV system by decreasing the activity of both hormone systems. In view of the potential interrelationship between aldosterone and PTH in patients with PHPT the findings from the EPATH study will facilitate our understanding of the putative benefits of eplerenone in patients with PTH excess, and the mechanisms between both hormone systems.

## Abbreviations

ABPM: 24-h Ambulatory Blood Pressure Monitoring; CV: Cardiovascular; CVD: Cardiovascular disease; HF: Heart failure; MR: Mineralocorticoid receptor; PHPT: Primary hyperparathyroidism; PTH: Parathyroid hormone; PTH/PTHrP receptor: PTH/PTH-related protein receptor; ZG: Zona glomerulosa.

## Competing interests

Supported by Pfizer (the study medication for the EPATH trial will be provided by Pfizer).

## Authors´ contributions

AT and SP designed the initial idea of this work. The manuscript has been read and approved by all authors.

## Pre-publication history

The pre-publication history for this paper can be accessed here:

http://www.biomedcentral.com/1472-6823/12/19/prepub
